# Humoral Dysregulation Associated with Increased Systemic Inflammation among Injection Heroin Users

**DOI:** 10.1371/journal.pone.0158641

**Published:** 2016-07-05

**Authors:** Michael S. Piepenbrink, Memorie Samuel, Bo Zheng, Brittany Carter, Christopher Fucile, Catherine Bunce, Michelle Kiebala, Atif A. Khan, Juilee Thakar, Sanjay B. Maggirwar, Diane Morse, Alexander F. Rosenberg, Norman J. Haughey, William Valenti, Michael C. Keefer, James J. Kobie

**Affiliations:** 1 Infectious Diseases Division, Department of Medicine, University of Rochester, Rochester, NY, United States of America; 2 School of Medicine, Howard University, Washington, DC, United States of America; 3 School of Medicine, Texas A&M University, Bryan, TX, United States of America; 4 Division of Allergy, Immunology, and Rheumatology, Department of Medicine, University of Rochester, Rochester, NY, United States of America; 5 Department of Microbiology and Immunology, University of Rochester, Rochester, NY, United States of America; 6 Departments of Psychiatry and Medicine, University of Rochester, Rochester, NY, United States of America; 7 Department of Neurology, Johns Hopkins University, Baltimore, MD, United States of America; 8 Trillium Health, Rochester, NY, United States of America; Tulane University, UNITED STATES

## Abstract

**Background:**

Injection drug use is a growing major public health concern. Injection drug users (IDUs) have a higher incidence of co-morbidities including HIV, Hepatitis, and other infections. An effective humoral response is critical for optimal homeostasis and protection from infection; however, the impact of injection heroin use on humoral immunity is poorly understood. We hypothesized that IDUs have altered B cell and antibody profiles.

**Methods and Findings:**

A comprehensive systems biology-based cross-sectional assessment of 130 peripheral blood B cell flow cytometry- and plasma- based features was performed on HIV-/Hepatitis C-, active heroin IDUs who participated in a syringe exchange program (n = 19) and healthy control subjects (n = 19). The IDU group had substantial polydrug use, with 89% reporting cocaine injection within the preceding month. IDUs exhibited a significant, 2-fold increase in total B cells compared to healthy subjects, which was associated with increased activated B cell subsets. Although plasma total IgG titers were similar between groups, IDUs had significantly higher IgG3 and IgG4, suggestive of chronic B cell activation. Total IgM was also increased in IDUs, as well as HIV Envelope-specific IgM, suggestive of increased HIV exposure. IDUs exhibited numerous features suggestive of systemic inflammation, including significantly increased plasma sCD40L, TNF-α, TGF-α, IL-8, and ceramide metabolites. Machine learning multivariate analysis distilled a set of 10 features that classified samples based on group with absolute accuracy.

**Conclusions:**

These results demonstrate broad alterations in the steady-state humoral profile of IDUs that are associated with increased systemic inflammation. Such dysregulation may impact the ability of IDUs to generate optimal responses to vaccination and infection, or lead to increased risk for inflammation-related co-morbidities, and should be considered when developing immune-based interventions for this growing population.

## Introduction

Injection drug use continues to be a major public health issue, with rapid growth in recent years. The rate of heroin use and overdose doubled in the United States between 2010 and 2012 [1, 2]. Numerous co-morbidities are associated with injection drug use including increased risk of cardiovascular disease, chronic kidney disease, gastrointestinal diseases, and infections [3–5]. Along with HIV and Hepatitis C, which are primarily transmitted in IDUs through sharing of contaminated needles, there is increased incidence of bacteremia [4] and soft-tissue injection site infections that include *Staphylococcus aureus*, Group A streptococci, and Clostridial infections [6–8]. In addition to increased exposure to pathogens, prolonged injection drug use has been associated with immune dysregulation that may diminish effective innate and adaptive immune responses [9].

Opiates, including heroin, can directly act on immune cells through opioid receptors, and result in decreased *in vitro* T cell proliferation and cytokine production [10], and decreased phagocytosis and chemotaxis of macrophages [11]. In mice, morphine has been shown to inhibit antibody responses [11–13]. Although heroin dominates illicit injection drug use, polydrug use is substantial among IDUs, including use of cocaine and methamphetamine [14, 15], which prevents clear extrapolation of the impact of opiates on the immune system in animal models to the complexity of IDUs, necessitating a direct and comprehensive assessment of the injection drug user population.

Several studies have reported elevated inflammatory mediators associated with IDUs, including serum IL-1β, IL-6, and IL-8 in methadone-maintained IDUs [16] [17], and increased dendritic cell and natural killer cell activation in IDUs that is associated with needle sharing [18]. These findings suggest that multiple mediators may be contributing to altered immune homeostasis in IDUs. Direct assessment of immune responsiveness in IDUs has been limited, although diminished antibody responses to vaccinations, including Hepatitis A and B have been observed in IDUs [19, 20]. Studies thus far have primarily measured only serum IgG antibody, and have not evaluated potential qualitative differences among the serum antibody response or direct evaluation of the B cells, which may influence vaccine efficacy, particularly for HIV [21].

The primary objective of preventative vaccine strategies against HIV is the induction of a persistent humoral response that mediates sterilizing immunity. Although modest, encouraging results of a short-lived reduction in infections in low-risk individuals were obtained in the RV144 HIV vaccine efficacy trial [22] for high-risk individuals, including IDUs, no substantial reduction in infection has been observed in HIV vaccine efficacy trials [23–25]. Furthermore, correlates of protection associated with RV144 included the IgG3 antibody response to a particular epitope (V1V2) on the HIV Envelope protein, and not the overall IgG antibody response to the vaccine [26], highlighting the qualitative subtleties that may determine HIV vaccine efficacy. Most mechanistic vaccine studies, including those for HIV vaccines, are primarily conducted with healthy subjects. This approach creates the vulnerability that advances in vaccine efficacy realized in healthy subjects may be difficult to translate to the IDU population, which is at high risk for HIV infection.

The impact of injection drug use on humoral responses is poorly defined. We sought to identify potential differences in the humoral profile of IDUs that could impact their immune responses and overall health. Through a cross-sectional assessment of peripheral blood B cells and plasma analytes, we have identified substantial alterations in IDUs that are associated with increased systemic inflammation, and which have the potential to influence the quality of the B cell and antibody response to vaccinations, infections, and contribute to inflammation-associated co-morbidities.

## Material and Methods

### Clinical samples and questionnaire

Peripheral blood samples were obtained from active IDUs recruited through the Trillium Health Syringe Exchange Program in Rochester, NY, during 2014. The IDUs were defined as having injected heroin within the previous 3 days and having a history of at least weekly heroin injection for a period of at least 3 months in duration within the previous 12 months. Healthy controls (HCs) with no history of substance abuse, no use of opiates (prescription or non-prescription), prescription stimulants, or illicit substances (excluding marijuana) within the previous 6 months were recruited at the University of Rochester Medical Center. All subjects were at least 110 lbs, 18–35 years old, HIV negative and Hepatitis C negative, and no history of immunization within the previous 2 weeks, as determined by self-report. All subjects provided signed, written informed consent. Competence and understanding of the informed consent was assessed throughout the process by study staff through a series of questions regarding study participation. Study subjects answered a series of questions regarding their demographics, health, substance use, sexual practices, and perspectives on vaccine research using a Research Electronic Data Capture (REDCap)–based questionnaire, hosted at the University of Rochester [27]. Peripheral blood was coded to blind the samples and was collected using CPT tubes (BD Biosciences, Franklin Lakes, NJ); peripheral blood mononuclear cells (PBMCs) and plasma were isolated and cryopreserved in a blinded manner as previously described [28] before subsequent analysis. All procedures and methods were approved by the University of Rochester Research Subjects Review Board.

### Flow cytometry

For B cell phenotypic analysis PBMCs were stained similar to as previously described [28, 29] with anti-CD19-APC-Cy7 (SJ25C1, BD), anti-CD20-AlexaFluor 700 (2H7, Biolegend, San Diego, CA), anti-CD3-PacificOrange (UCHT1, Invitrogen, Carlsbad, CA), anti-IgD-FITC (IA6-2, BD), anti-IgG-APC (G18-145, BD), anti-IgM-PE-Cy5 (G20-127, BD), anti-CD27-Qdot655 (CLB-27/1, Invitrogen), anti-CD21-V450 (B-ly4, BD), anti-CD14-PerCP-Cy5.5 (M5E2, BD), anti-CD138-biotin (B-A38, Abcam, Cambridge, MA), streptavidin Qdot800 (Invitrogen), anti-CD4-Qdot705 (S3.5, Invitrogen), anti-CD38-Qdot605 (HIT2, Invitrogen), anti-CD95-PE-Cy7 (DX2, Biolegend), anti-CD24-PE-AlexaFluor610 (SN3, Invitrogen), anti-CD183-PE (1C6/CXCR3, BD) and Live/Dead fixable aqua dead cell stain (Invitrogen). One-to-two million total events per sample were collected on an LSRII instrument (BD Biosciences). Staining and analysis performed in a blinded manner, linking sample with experimental group after gating was completed using FlowJo software (Treestar, Inc, Ashland, OR). Total PBMC were gated on lymphocytes using FSC and SSC. Live/Dead stain, anti-CD3, anti-CD4, and anti-CD14 were used to exclude dead cells, T cells, and monocytes respectively.

### Plasma antibody ELISAs and LAL

For detection of plasma total IgG and IgG subclasses ELISA plates were coated overnight with 1 μg/ml anti-human IgG (Jackson ImmunoResearch, 709-006-149, West Grove, PA) in PBS, blocked with 2% nonfat dry milk and 2% BSA in PBS for 1 h, then washed with 0.1% Tween 20 in PBS. Samples were serially diluted in triplicate in PBS containing 0.01% Tween 20 and 0.5% BSA, and incubated for 1h. Plates were washed, and binding was detected using anti-human IgG-HRP (Jackson), anti-human IgG1-HRP (Southern Biotech, 9052–05, Birmingham, AL), anti-human IgG2-HRP (Southern Biotech, 9070–05), anti-human IgG3-HRP (9210–05), or anti-human IgG4-HRP diluted 1:2000 in PBS with 0.1% Tween 20. Total plasma IgM and IgA were detected using 1 μg/ml anti-human IgM (Jackson, 109-006-129) with 1:2000 anti-human IgM-HRP (Jackson, 109-035-129), and 1 μg/ml anti-human IgA (Jackson, 109-005-011) with 1:2000 anti-human IgA-HRP (Jackson, 309-035-011). ELISA plates were coated with 0.5 μg/ml Tetanus toxoid (Calbiochem, San Diego, CA), 10 μg/ml LPS (Sigma- St. Louis, MO Aldrich), or 1 μg/ml Sm antigen (Arotec Diagnostics, Wellington, New Zealand) and detected with 1:2000 anti-human IgG-HRP to measure Tetanus toxoid, LPS, and Sm specific IgG plasma antibody respectively. ELISA plates were coated with 1 μg/ml HIV SF162 gp140 protein or 1 μg/ml HIV p24 protein (NIH AIDS Reagent Repository) and detected with either anti-human IgG-HRP or anti-human IgM-HRP to measure HIV-specific antibody. Anti-dsDNA IgG ELISA was performed, testing samples in duplicate using a commercial kit according to manufacturer's (Inova Diagnostics, Sand Diego, CA) recommendations. Plasma LPS was measured using the Kinetic-QCL chromogenic LAL assay (Lonza, Basel, Switzerland) according to manufacturer’s instructions.

### Plasma analyte multiplex assay

Each plasma sample was tested in duplicate in a blinded manner using 25 μl plasma plus the addition of 5 μl of heteroblock (Omega Biologicals, Bozeman, MT) to minimize non-specific interactions [30] with a MilliPlex Human Cytokine/Chemokine assay (Millipore, Billerica, MA, MPXHCYTO60KPMX42) according to manufacturer’s instructions. Values that were below limit of detection were adjusted to non-zero value (10, 1, 0.1, or 0.01) depending on lower limit of detection for individual analyte to enable statistical analysis.

### Ceramide profiling: Lipid extraction and LC/ESI/MS/MS analysis

Lipids were extracted from plasma using a modified Bligh and Dyer procedure as previously described [31]. The organic layer containing a crude lipid extract was dried using a nitrogen evaporator (Organomation Associates Inc., Berlin, MA, USA), and resuspended in pure methanol. Ceramides were detected by multiple reaction monitoring using a liquid chromatography coupled electrospray ionization tandem mass spectrometer (ESI/MS/MS) (API3000; AB Sciex Inc., Thornhill, ON, Canada) operated in positive mode. LC and MS/MS parameters have been previously described [32]. Slight differences in extraction efficiency and fluctuations in the efficiency of mass detection were normalized using a ceramide C12:0 internal standard (Avanti Polar Lipids, Alabaster, AL). Instrument efficiency was monitored daily, and at the end of the study individual plasma extracts were re-analyzed if the internal standard deviated more than 30% from the overall median internal standard value. Ceramide concentrations were determined by fitting the identified ceramide species to standard curves based on acyl-chain length. Ceramide standards C16:0, C18:0, C18:1 were purchased from Sigma. Ceramides C20:0, C24:0, C24:1 were purchased from Avanti Polar Lipids (Alabaster, AL). Palmitoyl-lactosyl ceramide C16:0-C16:0, stearoyl-lactosyl-ceramide C16:0-C18:0, lignoceryl-glucosyl-ceramide C16:0-C24:0, lignoceryl-galactosyl-ceramide C16:0-C24:0, and stearoyl-galactosyl-ceramide-sulfate C18:1-C24:0 were purchased from Matreya Inc. (Pleasant Gap, PA). Instrument control and quantitation were performed using Analyst 1.4.2, and MultiQuant software (AB Sciex Inc. Thornhill, Ontario, Canada).

### Statistical analysis

Univariate analysis of individual features was performed by the two-tailed Mann-Whitney test to compare groups using Prism 5.0 software (GraphPad Software, La Jolla, CA). No correction for multiple outcomes was performed due to the limited sample size and exploratory nature of the project. Heat maps, cluster analysis and principal component analysis were performed using Matlab (the Mathworks Inc., Natick MA).

Furthermore, a supervised machine learning classifier with wrapper method [33, 34] called support vector machine (SVM) recursive feature elimination [35, 36] was used to find small subsets of features that together have good predictive power to classify IDUs. In the first iteration, a SVM classifier was trained using the complete set of (216) features (**S1 File**). These features were then ranked based on their relative classification power in terms of their absolute weights. The feature with the smallest weight was removed iteratively, each time upon training a new SVM classifier. The performance of the classifier in each run was measured through 10-fold cross validation. IDU-23 was excluded from the training process because of its extreme outlier profile. IDU-27 was also excluded from the training process because of a missing “cell count” value. When these two subjects were independently tested on final SVM (built on top 10 features), the SVM was able to classify both of them accurately.

## Results

### Injection drug user population

Young adult HIV negative and Hepatitis C negative active IDUs (n = 19) were recruited through the local syringe exchange program. The majority of the IDU cohort was white (84%) and male at birth (68%) and similar in composition to the group of healthy controls (HCs) (**Table 1**). IDUs reported reduced self-perception of their overall health and satisfaction with their lives. Sexual risk behaviors did not significantly differ from HCs including past diagnosis of a sexually transmitted infection, number of sexual partners, or incidence of unprotected anal or vaginal intercourse. HIV testing rates were slightly higher among IDUs, as reported testing within the previous 6 months was 47% in IDUs vs 28% in HCs, and 68% in IDUs vs 50% in HCs within the previous year; likely driven by point-of-care HIV testing offered at the syringe exchange. Encouragingly, the majority (73%) of IDUs indicated they were agreeable to participating in a hypothetical future HIV vaccine trial, and participating in a hypothetical future research study that requires 10 visits (74%).

**Table 1:** Participant Characteristics.

	Healthy Control Group (n = 19)	Injection Drug User Group (n = 19)	
Age (yrs)	24.3 (18–35)	28.4 (21–35)	**p = 0.014**
Female (at birth)	42%	32%	p = 0.2425
Transgender	5%	5%	p = 1
Black	11%	5%	p = 0.1913
White	79%	84%	p = 0.4667
Body mass index	29.4 (19–47)	26.1 (21–38)	p = 0.1409
“I seem to get sick a little easier than other people” (1 = definitely true, 5 = definitely false)	3.8 (3–5)	3.1 (2–5)	**p = 0.005**
“In general how satisfied are you with your life?” (very dissatisfied to very satisfied, % satisfied to very satisfied)	95%	26%	**p<0.0001**
≤ $15,000 annual income	47%	74%	**p = 0.0002**
College graduate	58%	11%	**p<0.0001**
Men who have sex with men (of males)	50%	8%	**p<0.0001**
# of sexual partners in last 6 months	3.2 (0–30)	3.7 (0–42)	p = 0.8603
Unprotected anal sex in last 6 months (not with main partner)	5%	5%	p = 1
Sex for money, drugs, gifts, or services	5%	5%	p = 1
New STI in last 6 months	5%	0%	p = 0.0594
Ever diagnosed with STI	21%	26%	P = 0.5050
HIV test w/in last 3 months	17%	37%	**p = 0.0023**
HIV test w/in last 6 months	28%	47%	**p = 0.0084**
HIV test w/in last year	50%	68%	**p = 0.0143**
Willing to participate in future HIV vaccine trial: agree or strongly agree	68%	73%	p = 0.5353
Willing to participate in a future research study that requires 1 visit: agree or strongly agree	100%	84%	**p<0.0001**
Willing to participate in a future research study that requires 10 visits: agree or strongly agree	63%	74%	p = 0.128

All IDUs had injected heroin within the preceding 72 hours of participation, and 56% reported to had used heroin every day for the preceding 3 months (**Table 2**). The combined use of heroin and cocaine was predominant, with 89% of IDUs reporting having injected cocaine within the preceding 30 days. Of the IDUs, 44% indicated they were intoxicated for most of the day for at least the majority of the days within the preceding 90 days. Occasional sharing of syringes in the last 30 days was reported by 20% of the IDUs, and the remainder indicated they had not shared needles in the past 30 days.

**Table 2:** Substance Use Characteristics.

	Healthy Control Group (n = 19)	Injection Drug User Group (n = 19)	
Tobacco use (any in last year)	24%	74%	**p< 0.001**
Alcohol (at least 15 out of last 90 days)	16%	21%	p = 0.467
Marijuana (at least 15 out of last 90 days)	0%	37%	**p< 0.001**
Drunk or high most of the day (at least 1 day out of last 90 days)	21%	78%	**p< 0.001**
Drunk or high most of the day (at least 16 out of last 90 days)	0%	61%	**p< 0.001**
Drunk or high most of the day (at least 46 out of last 90 days)	0%	44%	**p< 0.001**
Crack cocaine (at least 16 out of last 90 days)	NA	26%	
Non-crack cocaine (at least 16 out of last 90 days)	NA	56%	
Heroin (at least 16 out of last 90 days)	NA	100%	
Heroin (everyday of last 90 days)	NA	56%	
Injected cocaine (at least once in last 30 days)	NA	89%	
Most recent injection was both heroin and cocaine	NA	42%	
Never have shared needles (in last 30 days)	NA	80%	
Occasionally have shared needles (in last 30 days)	NA	20%	
Often or all the time shared needles (in last 30 days)	NA	0%	

### Increased total B cells and B cell activation among IDUs

**Fig 1:**
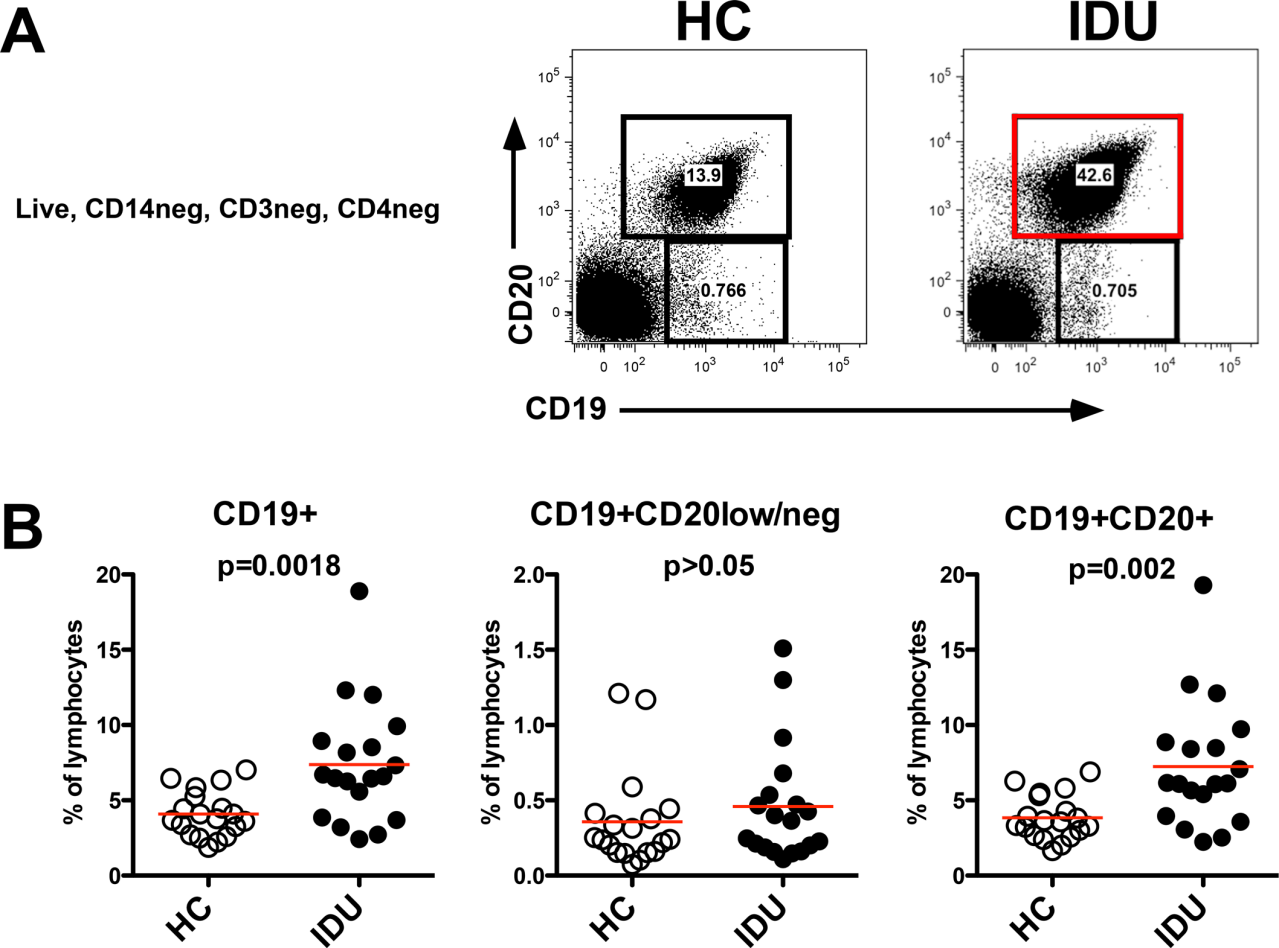
Increased total peripheral blood B cells in IDUs. PBMC were analyzed by flow cytometry. (**A**) Representative plots gated on live, CD14-CD3-CD4- lymphocytes, the gate is colored red to highlight the expanded CD19+CD20+ total B cell population in IDUs. (**B**) Frequency of CD19+ (live, CD14-CD3-CD4-) populations among lymphocytes defined by FSC and SSC, each symbol is an individual subject, the red lines indicate mean. The singular CD19+ population is the combination of both the CD19+CD20low/neg population and the CD19+CD20+ population. Flow cytometry was conducted once per sample.

**Fig 2:**
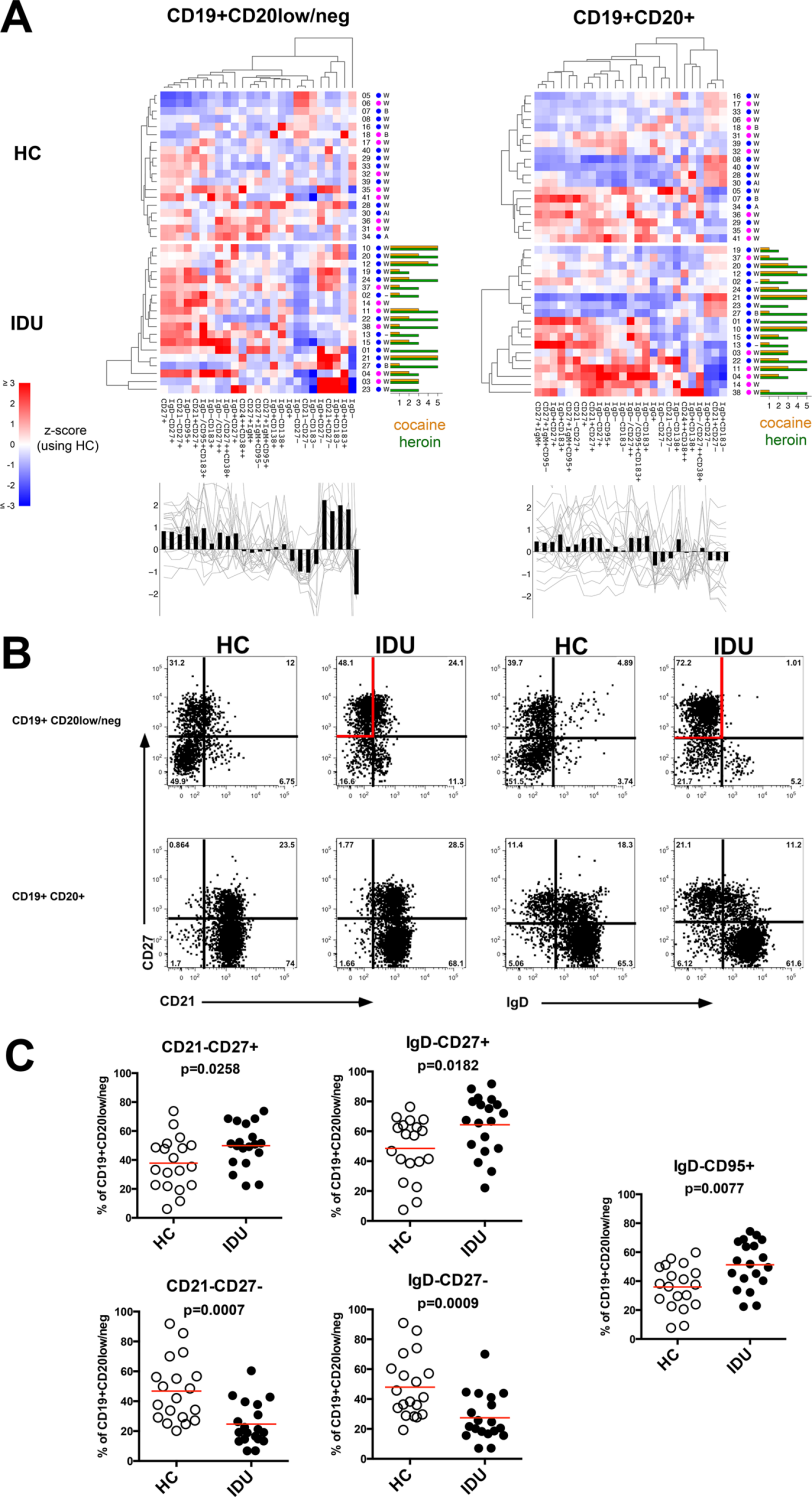
B cell phenotypic profile. (**A**) B cell profiles for live, CD14-CD3-CD4- CD19+CD20low/neg (left) and CD19+CD20+ (right). Heat maps show data corresponding to a particular B cell subset (column) and subject (row). Colors correspond to z-scores computed separately for each subset using the mean and standard deviation of the HCs. Both subsets and subjects were clustered hierarchically based on Euclidean distance and complete linkage, although HCs and IDUs were clustered separately. Sample ID, gender (F = magenta, M = blue) and race (W = white, B = black, A = Asian, AI = American Indian,— = unknown) are shown just to the right of each heat map. Cocaine and heroin usage is shown as a bar graph to the right of the injection drug user sample heat maps. Bar plot below each heat map shows the mean z-score for each subset for the IDUs samples only (z-score based on mean, standard deviation of HCs only). Gray traces represent data from individual IDU samples. (**B**) Representative plots gated on live, CD14-CD3-CD4- CD19+CD20low/neg or CD19+CD20+ B cell populations, the gates are colored red to highlight expanded CD19+CD20low/neg CD27+ subsets in IDUs. (**C**) Frequency of select CD19+CD20low/neg subsets, each symbol represents an individual subject, the red lines indicates mean.

As B cells are critical for protective immunity we investigated the phenotypic profile of the peripheral blood B cell compartment for altered homeostasis. There were no significant differences in total lymphocyte count, frequency of CD14+ monocytes, or CD4+ T cells among the groups (not shown) however IDUs had approximately 2-fold higher frequency of total B cells, defined as CD19+ as compared to HCs (8.6% vs. 4.4%, p<0.005) (**Fig 1A and 1B**). This observation was most pronounced in the CD19+CD20+ B cell population (7.2% vs 3.8%, p<0.005), that is known to include nearly all peripheral blood B cell subsets. No significant difference was observed in the frequency of the CD19+CD20low/neg B cell population. The CD19+CD20low/neg population is primarily described as being enriched for plasmablasts, which are active antibody secreting cells, or pre-plasmablasts [37, 38]. A comprehensive phenotypic profiling of the B cell subsets (**Fig 2)** did not reveal significant phenotypic differences within the CD19+CD20+ B cell population, although there was a notable 40% increase in IgD+CD183(CXCR3)+ B cells among IDUs (p = 0.0935), suggesting there may be a low-level chronic inflammatory phenotype in IDUs [39–41].

Although the frequency of the CD19+CD20low/neg B cell population was not significantly different between groups, numerous phenotypic differences were apparent within this population. Within the CD19+CD20low/neg B cell population, IDUs exhibited a 30% increase in CD21-CD27+ (p<0.05), 30% increase in IgD-CD27+ (p<0.05), with a reciprocal 50% decrease in CD21-CD27- (p<0.005) and 40% decrease in IgD-CD27- (p<0.005), in addition to a 40% increase in IgD-CD95+ (p<0.005) (**Fig 2B and 2C**). The CD19+CD20low/neg plasmablast population has previously been defined by CD38 or CD138 expression [28, 38, 42], however, no significant differences were observed between groups among CD19+CD20low/neg CD27+CD38+ or CD138+ potential plasmablast populations (**Fig 2A**). The increased CD27 and CD95 expression among the CD19+CD20low/neg is consistent with an activated B cell phenotype in IDUs.

### Skewed plasma antibody profile among IDUs

**Fig 3:**
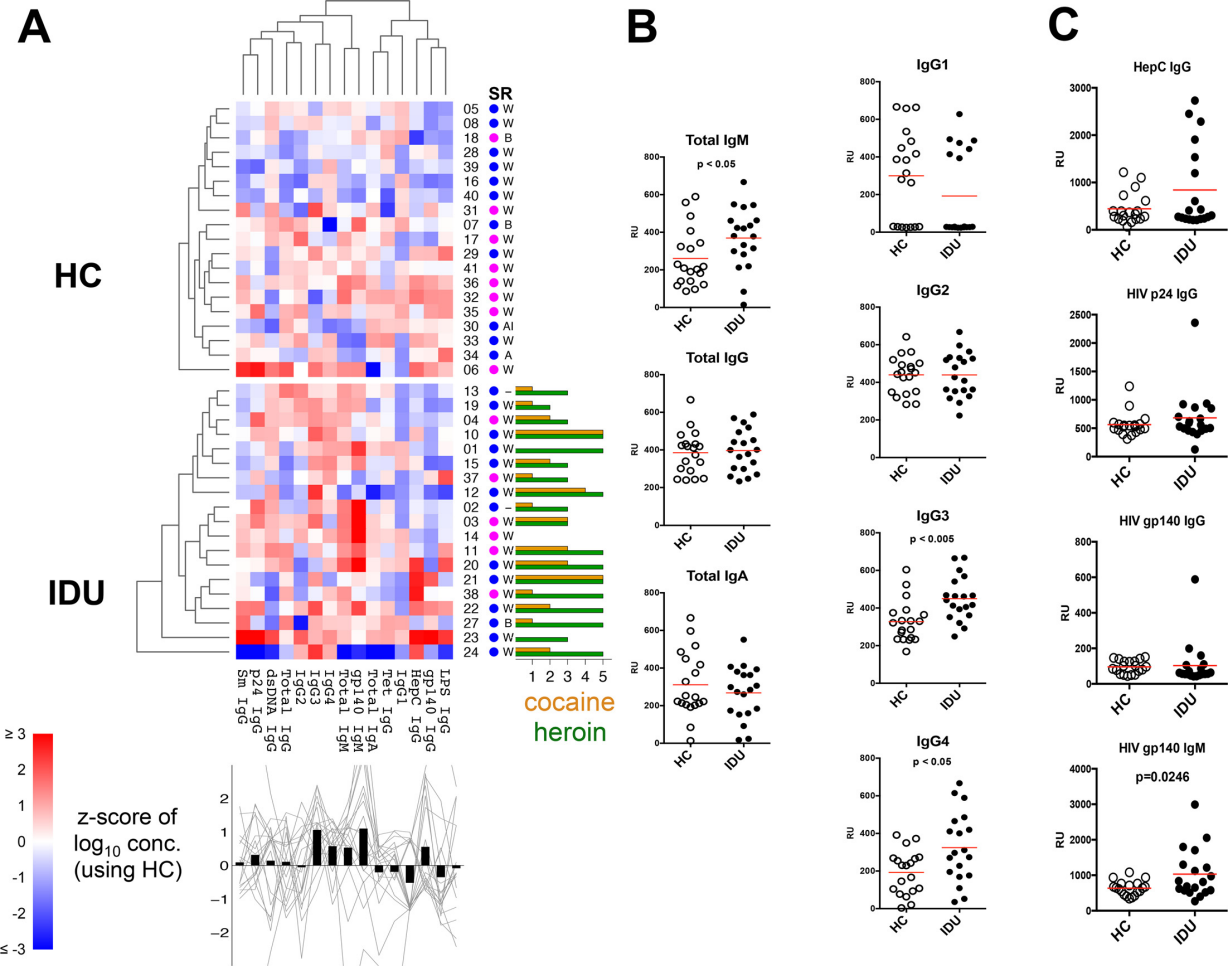
Plasma antibody profile. Relative concentrations of plasma antibody species was determined by ELISAs. (A) Antibody profiles are shown as a heat maps similar to Fig 1. Antibody concentrations were log-transformed prior to computing z-scores. (B) Plasma concentrations of total antibody isotypes and IgG subclasses and (C) select antigen-specific antibodies. Each symbol represents an individual subject, the red lines indicate mean.

Observing such prominent differences in the phenotype of the B cells in IDUs, we evaluated if these alterations extended to the plasma antibody compartment. Comprehensive profiling of plasma antibodies revealed gross differences between IDUs and HCs (**Fig 3A**). IDUs exhibited significantly higher total IgM concentration (p<0.05), and although total IgG concentrations were comparable between groups, IDUs had significantly higher IgG3 (p<0.005) and IgG4 (p<0.05) subclasses (**Fig 3B**). No significant difference in total IgA concentration was apparent. This skewing of plasma antibody profiles suggests a higher frequency of subclinical infections or ongoing inflammation in IDUs [43].

No significant differences in the incidence of auto-reactive IgG antibody (anti-Sm, anti-dsDNA) were observed (**Fig 3A**), suggesting that self-tolerance was being maintained in IDUs. Several IDUs had increased HepC-specific IgG that may suggest previous exposure to the virus. We next examined if there was any serum antibody reactivity to HIV; specifically we did not observe any significant differences in anti-HIV p24 IgG or anti-HIV gp140 IgG between the two groups; however, IDUs had significantly higher (p<0.05) HIV gp140 IgM antibody titers (**Fig 3C**), which may suggest increased exposure to HIV within this population, and may reflect a history of exposure that did not result in HIV infection. Only one subject (IDU-23) exhibited anti-HIV p24 IgG and anti-HIV gp140 IgG antibody reactivities that were substantially higher than the group of HCs, this subject also had numerous elevated serum antibodies including anti-HepC IgG, -LPS IgG, -Sm IgG, and -dsDNA IgG, which could indicate undiagnosed HIV infection or high levels of poly-reactive serum antibodies.

### Increased systemic inflammatory mediators in IDUs

**Fig 4:**
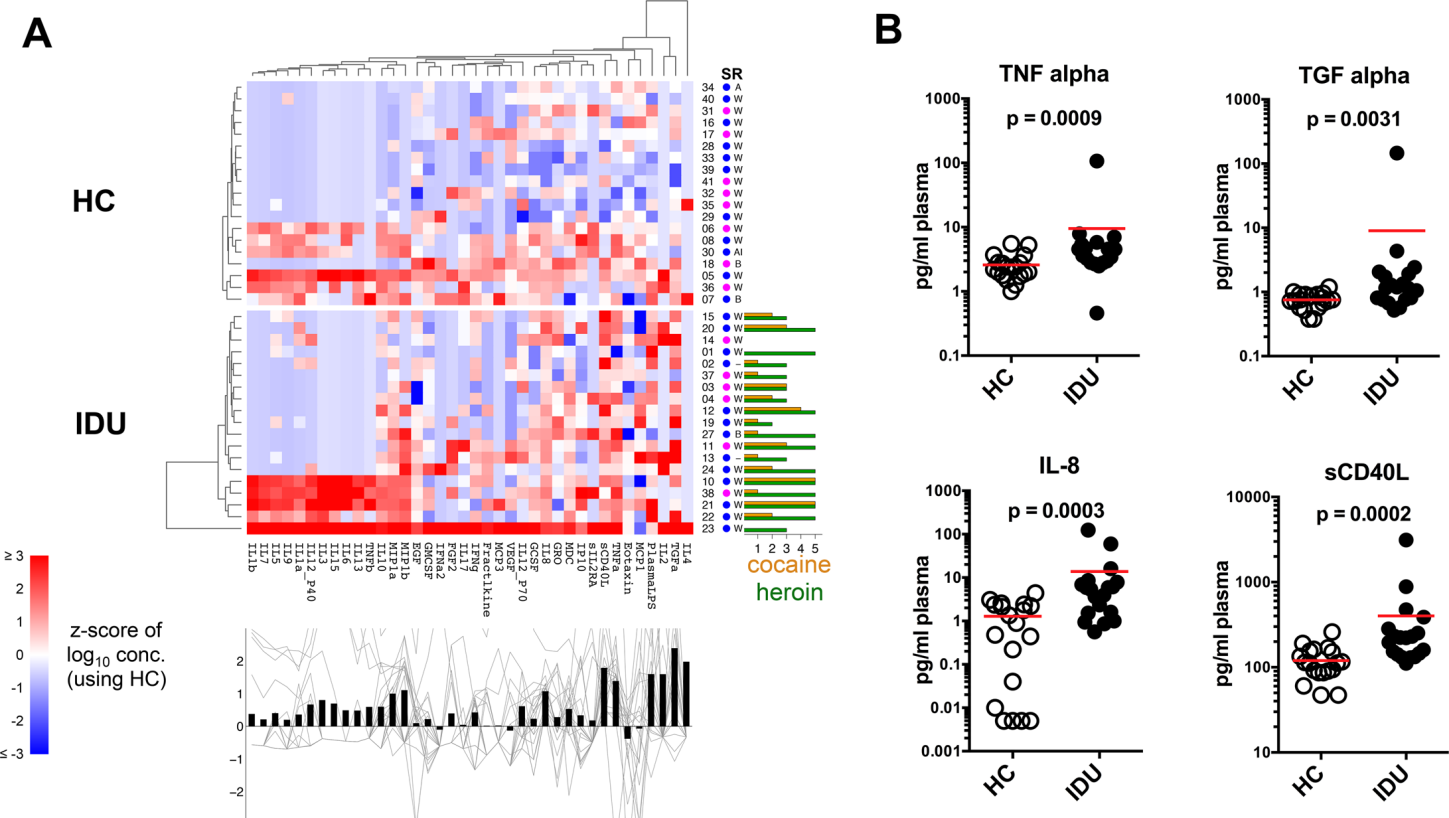
Plasma analyte profile. Plasma cytokines, chemokines and growth factors were measured by Milliplex Immunoassay and LPS was measured by a limulus assay. (A) Analyte profiles are shown as a heat map similar to above. Analyte concentrations were log-transformed prior to computing z-scores. (B) Select plots of individual analytes, each symbol represents an individual subject, the red lines indicate mean.

To assess potential systemic alterations in innate immune mediators, an extensive panel of plasma cytokines, chemokines and soluble factors was measured (**Fig 4**). The group of IDUs had significantly increased plasma TGF-α (~10-fold, p<0.005; an EGFR ligand), TNF-α (~5-fold, p<0.005; a potent inflammatory cytokine); IL-8 (~10-fold, p<0.001; a chemokine important for neutrophil migration, induction of phagocytosis and angiogeneisis), and sCD40L (~5-fold, p<0.0005; a potent inflammatory mediator produced primarily by platelets, which induces proliferation and class-switching of B cells [44]) (**Fig 4B**). We also found increased levels of plasma LPS (endotoxin) evident in several IDUs (p<0.01) (**Fig 4A**), suggestive of bacterial infection or increased gut permeability [45, 46]. These results are consistent with a chronic systemic inflammatory phenotype in IDUs.

### Altered ceramide profile in IDUs

**Fig 5:**
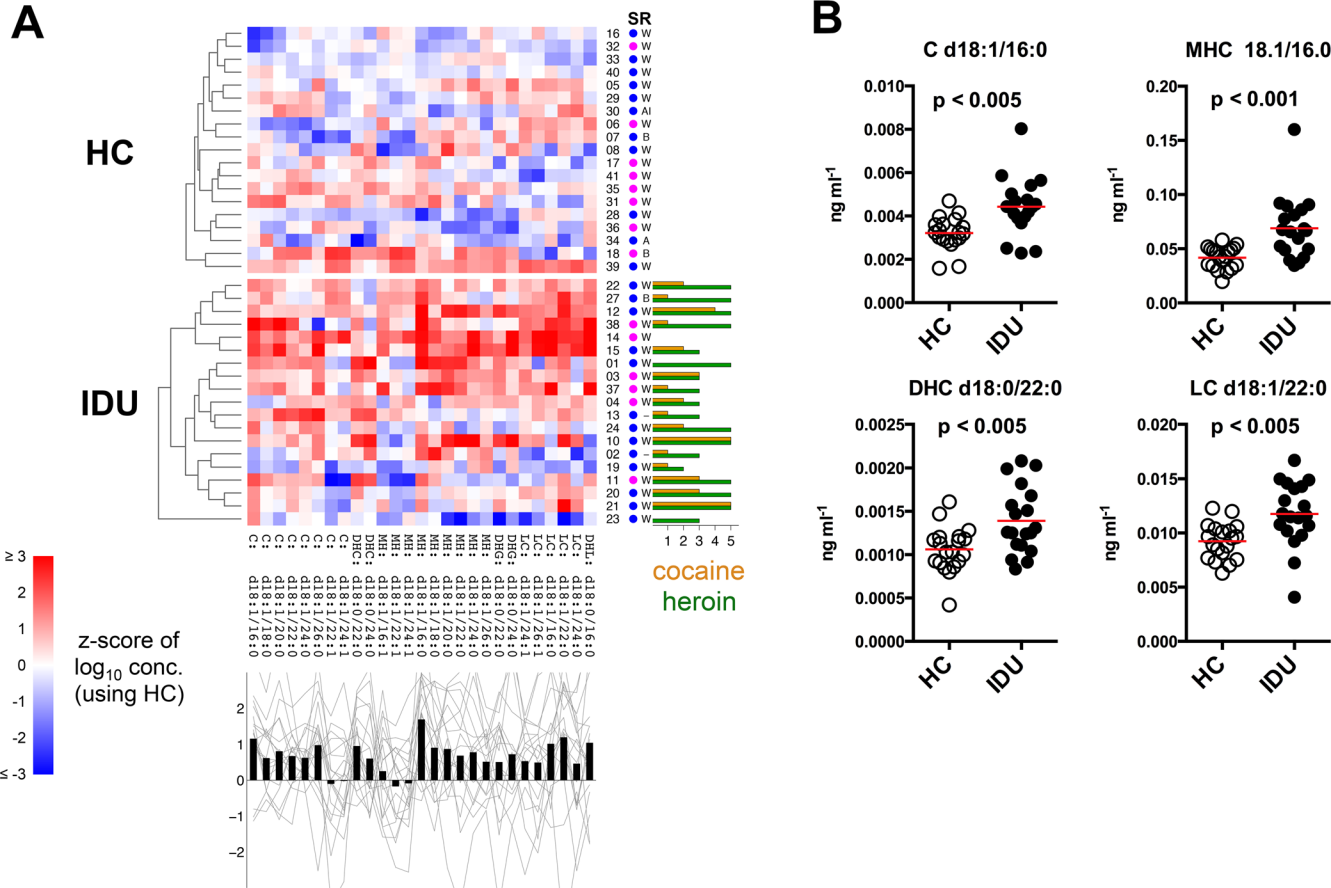
Plasma ceramide profile. Plasma ceramide species were measured by LC/ESI/MS/MS analysis. (A) Ceramide profiles are shown as a heat map similar to above. Ceramide concentrations were log-transformed prior to computing z-scores. Ceramide profile elements (columns) were not clustered. (B) Select plots of individual ceramide species, each symbol represents an individual subject, red lines indicate mean.

Ceramides are bioactive lipids present in the circulation as components of lipoprotein complexes and exosomes [47–50]. This class of sphingolipid regulates intra- and intercellular signaling associated with inflammation, cellular growth, proliferation, differentiation, senescence, and apoptosis [51]. At low levels, ceramides are important for injury-induced cytokine production and for activating protein phosphatases and kinases involved in stress-related signaling cascades [51]. However, at high levels, ceramides inhibit cell division and induce cellular dysfunction and apoptosis. Ceramide metabolism is both impacted by, and contributes to inflammation [52, 53]. We measured plasma levels of simple and complex ceramides and found considerable up-regulation of numerous ceramide species in the IDUs (**Fig 5A**) that included significant increases of ceramide d18:1/16:0 (p<0.005), monohexosylceramide 18.1/16.0 (p<0.001), dihydroceramide d18:0/22:0 (p<0.005), and lactosylceramide d18:1/22:0 (p<0.005) (**Fig 5B**) consistent with inflammation in IDUs.

### Machine learning identifies major distinguishing features of IDUs

**Fig 6:**
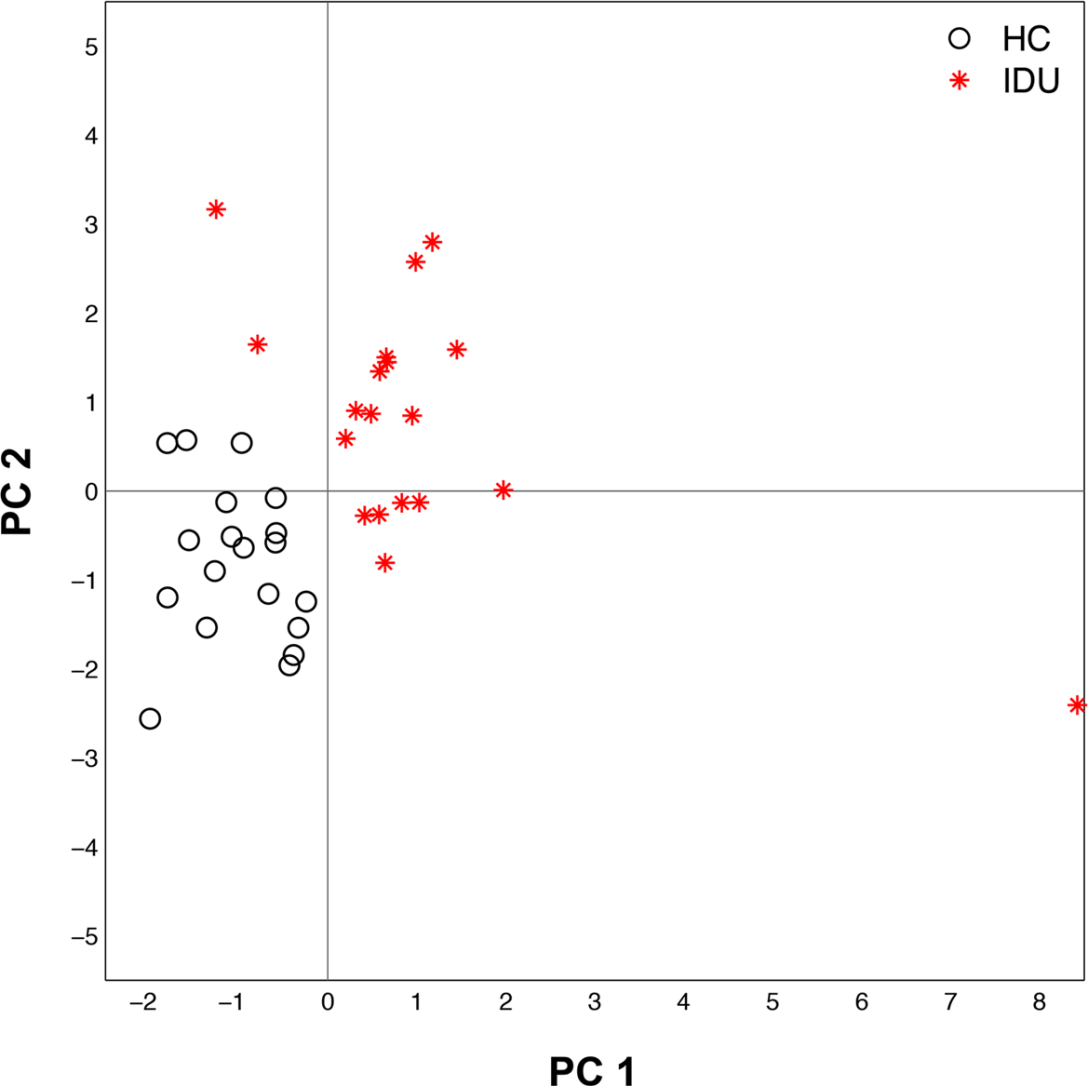
Principal component analysis of features identified by machine learning pipeline. Principal component analysis (PCA) of 19 HCs (black circles) and 19 IDUs (red asterisks) based on ten features from the various assays identified by machine learning (CD19+CD20low/negIgD-CD27-, MIP-1β, gp140 IgM, IgG4, CD19+CD20+, sCD40L, TGF-α, MH: d18:1/16:0, TNF-α and MH: d18:1/22:0). PCA was computed on per-feature z-scores (based on all 38 samples) of log transformed data (except for B cell subset frequencies which were not log-transformed).

To distill the features that best distinguish IDUs from HCs, a machine learning multivariate analysis approach was utilized that incorporated all measured features including cell phenotyping, plasma antibody, plasma cytokine/chemokine/factor, and plasma ceramide profiling data. A supervised recursive feature elimination approach was utilized to identify the top ten features that when combined, classified the samples as IDU or HC with 100% accuracy using 10-fold cross-validation (**Table 3**). IDUs were classified as having increased CD19+CD20+ B cells, MIP1-β, sCD40L, TGF-α, TNF-α, anti-HIV gp140-specific IgM, total IgG4, MHC d18.1/16:0, and MHC d18.1/22:0 with decreased IgD-CD27-CD19+CD20low/neg cells as compared to HCs. Principal components analysis that considered only these ten selected features completely segregated samples as either IDU or HC (**Fig 6**). These results demonstrate the breadth of systemic alterations present in IDUs, and resolve a core set of features that may be of value to monitor in future investigations of IDUs.

**Table 3:** Features Identified by Machine Learning Analysis.

Ranked using SVM weights	Feature	HC (mean)	IDU (mean)	P (univariate)
1	sCD40L (pg/ml)	120.2	249.5	0.0004
2	IgG4 (RU)	192.7	308.6	0.0310
3	CD19+CD20+ (% of lymphocytes)	3.8	7.2	0.0036
4	TGF-α (pg/ml)	0.8	1.4	0.0056
5	TNF-α (pg/ml)	2.6	4.1	0.0016
6	MIP-1β (pg/ml)	4.4	13.2	0.0330
7	IgD-CD27- (% of 19+20low)	48.0	27.6	0.0015
8	MonoHex Ceramide d18.1/16:0 (ng/ml)	0.04	0.07	<0.0001
9	gp140_IgM (RU)	634.9	1055.0	0.0529
10	MonoHex Ceramide d18:1/22:0 (ng/ml)	0.01	0.012	0.0269

## Discussion

Our comprehensive humoral-centric immunological profiling study revealed dramatic dysregulation in active heroin IDUs. This was apparent at the gross level as increased total B cells and total plasma IgM in the IDUs, and finer analysis revealed altered B cell phenoytpes; most prominent within the minor CD19+CD20low/neg compartment with increased CD27+ and CD95+ subsets, and increased plasma IgG3 and IgG4 subclasses in IDUs. Together, these differences suggest greater immune activation at steady state in IDUs, a conclusion that is supported by significant increases in numerous plasma inflammatory mediators including sCD40L and TNF-α. The underlying factors driving inflammation in IDUs are uncertain and warrant further investigation.

Increased inflammation in IDUs could be a consequence of numerous factors. Opioids have primarily been associated with decreased immune activation in studies of acute treatment and cessation of use, however increased plasma IL-1β, IL-6, IL-8, IL-10, and TNF-α were observed in heroin addicts undergoing methadone treatment as compared to healthy controls [16]. It has been postulated by others that in the setting of chronic opioid use, lymphocytes and other cell types may no longer be sensitive to opioids or perhaps could be hyper-responsive to opioids as a result of altered mu receptor expression and signaling [54, 55]. The majority (89%) of our cohort of IDUs reported frequent cocaine use, which has been associated with increased immune activation and inflammation in several studies [56–59]. The concomitant use of both heroin and cocaine among IDUs, drugs that have been reported to have grossly opposite immunological impacts, highlights the necessity of directly assessing this complex population. In studies by Tomescu et al. [18, 60], increased inflammation, including NK cell activation, plasma IP-10, and dendritic cell maturation were reported in IDUs, although mostly confined to needle-sharing IDUs and not significantly elevated in non-sharing IDUs, suggesting that inflammation may be driven by factors associated with the sharing of needles, such as increased exposure to blood-borne pathogens or allo-antigens. Our findings of increased inflammation in non-needle sharing IDUs do not necessarily contradict those of Tomescu et al. Perceived differences may be a result of our larger sample size and non-identical outcome measures, and together suggest that the immune dysregulation, such as increased total B cells and skewed plasma IgG subclasses we observed, may be further exacerbated in needle-sharing IDUs.

The detection of LPS in the plasma of several IDUs suggests increased bacterial exposure. This is consistent with increased bacterial infections, including sepsis, in IDUs [45], and may contribute to TLR-driven inflammation. Increased plasma LPS is commonly observed in HIV-infected patients and high-risk subjects, and has frequently been associated with, and suggested to contribute to increased systemic inflammation [61–65]. Plasma LPS in HIV-infected patients is suggested to be a consequence of microbial translocation, and is further elevated in HIV+ IDUs compared to HIV+ non-IDUs [46]. Thus, we speculate that increased LPS in our IDUs cohort may be the consequence of both exogenous bacterial exposure and microbial translocation.

Recent advances in lipidomics have begun to reveal disease, aging, and infection associated alterations in ceramides. Increased plasma ceramides have been associated with coronary artery disease, alcohol abuse, chronic kidney disease, diabetes, HIV-Associated Neurocognitive Disease, Alzheimer’s disease Parkinson’s disease, and depression [32, 66–73]. Elevated plasma ceramide in IDUs highlights potential intersections of injection drug use with declines in physiological and cognitive function [4, 74–80], although these associations were not directly tested in this study. Likewise, it is unclear if ceramides can directly impact the function of B cells, and these observations warrant further investigation. Interestingly, ceramides can facilitate viral entry, including HIV attachment, and the particular ceramide composition of virions generated by budding through the cell membrane can impact viral function [81], suggesting that up-regulation of ceramides may facilitate viral infections in IDUs.

The up-regulation of inflammatory mediators in IDUs likely act directly on their B cells; for example sCD40L and TNF-α may drive B cell expansion, perhaps in a polyclonal fashion [82], but may also exacerbate responses to insulting antigens (e.g. drug contaminants, allo-antigens, microbes) contributing to IgG3 and IgG4 skewing. This process may be amplified by indirect actions of TNF-α and IL-8 on accessory cells that shape the B cell response, such as T cells, antigen presenting cells, and neutrophils. Despite the remarkable 2-fold expansion of CD19+CD20+ B cells in IDUs, substantial phenotypic differences were not apparent, with the exception of increased IgD+CD183(CXCR3)+ in IDUs. CXCR3 expression on B cells has been suggested to indicate migration to sites of inflammation [39, 40], and increased CXCR3+ B cells have been observed in patients with chronic inflammation, including those with rheumatoid arthritis and systemic lupus erythematous [41], indicating their increase in IDUs is consistent with an increased inflammatory state. The CD19+CD20low/neg compartment appears to be most sensitive to the increased inflammatory state of the IDUs, perhaps in-part driven by its relative small abundance (~0.5% of PBMC). This B cell compartment and its subsets including the CD19+CD20low/negCD27+ cells which are increased in IDUs, should be evaluated further for antibody secretion to determine if they are functionally pre-plasmablasts that have been described previously in other contexts [37, 75, 83, 84]. Additionally, immunoglobulin repertoire analysis may reveal the extent to which B cell alterations in IDUs are a function of antigen-driven or antigen-independent (e.g. TLR) expansion. The limited scope of our study did not directly address whether injection drug use alters the functions of B cells, such as their ability to proliferate, differentiate, or produce cytokines in response to stimulation. However, chronic inflammation may drive an exhausted-like immune response similar to that observed in chronic infections [85, 86], or conversely hyper-responsiveness, similar to what we observed in early-stage rheumatoid arthritis patients [28]. Both scenarios are likely to impact the characteristics and efficacy of the B cell response to pathogens and vaccines in IDUs.

HIV is a major infectious disease threat for IDUs, and the substantial B cell alterations we have observed in IDUs suggest they could have qualitatively distinct responses to HIV vaccine candidates, potentially compromising the efficacy of future HIV vaccine strategies in this population. The rigorous comparison by Yates et al. [26] of VAX003, the largest HIV vaccine efficacy trial conducted thus far in IDUs to RV144, which was conducted in predominantly low-risk subjects, may be the most compelling yet underappreciated example of how IDUs may generate qualitatively distinct HIV vaccine responses. Both trials utilized the same AIDSVAX gp120 protein immunogen, although RV144 included a canarypox prime (ALVAC) and VAX003 only repetitive AIDSVAX immunizations. Yates et al. [26] examined the V1V2-specific IgG3 response rate, which correlated with a decreased risk of infection in RV144. RV144 participants had a higher response rate than VAX003 recipients (~60% vs ~20%-40%), however, those who did respond in VAX003 had a significantly higher magnitude of response than the RV144 responders [26]. Also, >90% of VAX003 participants had a gp120-specific IgG4 response, in contrast to <20% of RV144 participants, which was suggested to be a consequence of the two additional AIDSVAX boosts that the VAX003 participants received [26]. In light of our findings of significantly higher total IgG3 and IgG4 in IDUs, another non-exclusive and complementary interpretation is that IDUs have a greater predisposition to generate vaccine-specific IgG3 and IgG4 responses, which we are actively investigating. A major detriment to exacerbated IgG3 and IgG4 responses is the short half-life of IgG3 and the inability of IgG4 to activate complement [43], which could dramatically impact the durability and functional activity of the humoral response, and may be a potential contributor to the increase in re-occurring infections observed among IDUs.

A common concern for detailed immune profiling studies on limited numbers of samples, such as ours is the extent to which false discovery or multiple outcomes can potentially accentuate biological features that are actually random and not authentic differences between groups. Our combined use of conventional univariate analysis and multivariate machine learning analysis revealed both features that have been previously reported to be impacted by injection drug use and novel features not previously identified. A follow-up study focused on these core features, that included both larger sample sizes and a longitudinal component would be ideal to thoroughly ascertain their true modulation in IDUs.

The elevated systemic inflammation in IDUs that we observed is consistent with previous studies [16, 17, 87–89], and may contribute to co-morbidities within this population. Chronic inflammation is associated with cardiovascular disease, kidney disease, accelerated aging, and neurodegenerative diseases, all of which are increased among IDUs [4, 74–79]. Although underlying HIV or Hepatitis C infections may also contribute to systemic inflammation and these co-morbidities, our results indicate that other factors may contribute. Not previously well-appreciated was the extent of humoral dysregulation among IDUs. In addition to potentially contributing to inflammation-mediated co-morbidities, the humoral alterations described here may have significant consequences for the protection of IDUs from pathogens, including their ability to generate effective responses to vaccines. Investigations into the impact of anti-inflammatory interventions in IDUs are warranted, and may yield broad health benefits for this growing population.

## Supporting Information

S1 File SVM Features. List of features used in SVM analysis.
(XLSX)

S2 File Data. Contains study data.
(XLSX)

## References

[1] Rudd RA, Paulozzi LJ, Bauer MJ, Burleson RW, Carlson RE, Dao D, et al Increases in heroin overdose deaths—28 States, 2010 to 2012. MMWR Morb Mortal Wkly Rep. 2014;63(39):849–54. . 25275328 PMC4584873

[2] Dart RC, Severtson SG, Bucher-Bartelson B. Trends in opioid analgesic abuse and mortality in the United States. N Engl J Med. 2015;372(16):1573–4. 10.1056/NEJMc1501822 . 25875268

[3] Fugelstad A, Annell A, Agren G. Long-term mortality and causes of death among hospitalized Swedish drug users. Scand J Public Health. 2014;42(4):364–9. 10.1177/1403494814525006 . 24608092

[4] Buettner M, Toennes SW, Buettner S, Bickel M, Allwinn R, Geiger H, et al Nephropathy in illicit drug abusers: a postmortem analysis. Am J Kidney Dis. 2014;63(6):945–53. 10.1053/j.ajkd.2014.01.428 . 24823296

[5] Dettmeyer R, Friedrich K, Schmidt P, Madea B. Heroin-associated myocardial damages—conventional and immunohistochemical investigations. Forensic Sci Int. 2009;187(1–3):42–6. 10.1016/j.forsciint.2009.02.014 . 19304418

[6] Lavender TW, McCarron B. Acute infections in intravenous drug users. Clin Med (Lond). 2013;13(5):511–3. 10.7861/clinmedicine.13-5-511 . 24115713 PMC4953807

[7] Gonzales y Tucker RD, Frazee B. View from the front lines: an emergency medicine perspective on clostridial infections in injection drug users. Anaerobe. 2014;30:108–15. 10.1016/j.anaerobe.2014.09.005 . 25230330

[8] Murphy EL, DeVita D, Liu H, Vittinghoff E, Leung P, Ciccarone DH, et al Risk factors for skin and soft-tissue abscesses among injection drug users: a case-control study. Clin Infect Dis. 2001;33(1):35–40. 10.1086/320879 . 11389492

[9] Kaushik KS, Kapila K, Praharaj AK. Shooting up: the interface of microbial infections and drug abuse. J Med Microbiol. 2011;60(Pt 4):408–22. 10.1099/jmm.0.027540-0 . 21389334

[10] Sacerdote P, Franchi S, Gerra G, Leccese V, Panerai AE, Somaini L. Buprenorphine and methadone maintenance treatment of heroin addicts preserves immune function. Brain Behav Immun. 2008;22(4):606–13. 10.1016/j.bbi.2007.12.013 . 18294814

[11] McCarthy L, Wetzel M, Sliker JK, Eisenstein TK, Rogers TJ. Opioids, opioid receptors, and the immune response. Drug Alcohol Depend. 2001;62(2):111–23. . 11245967 10.1016/s0376-8716(00)00181-2

[12] Taub DD, Eisenstein TK, Geller EB, Adler MW, Rogers TJ. Immunomodulatory activity of mu- and kappa-selective opioid agonists. Proc Natl Acad Sci U S A. 1991;88(2):360–4. 1846441 10.1073/pnas.88.2.360 PMC50810

[13] Jamali A, Roostaee MH, Soleimanjahi H, Ghaderi Pakdel F, Bamdad T. DNA vaccine-encoded glycoprotein B of HSV-1 fails to protect chronic morphine-treated mice against HSV-1 challenge. Comp Immunol Microbiol Infect Dis. 2007;30(2):71–80. 10.1016/j.cimid.2006.10.002 . 17126902

[14] Meacham MC, Rudolph AE, Strathdee SA, Rusch ML, Brouwer KC, Patterson TL, et al Polydrug Use and HIV Risk Among People Who Inject Heroin in Tijuana, Mexico: A Latent Class Analysis. Subst Use Misuse. 2015;50(10):1351–9. 10.3109/10826084.2015.1013132 . 26444185 PMC4786000

[15] Kuramoto SJ, Bohnert AS, Latkin CA. Understanding subtypes of inner-city drug users with a latent class approach. Drug Alcohol Depend. 2011;118(2–3):237–43. 10.1016/j.drugalcdep.2011.03.030 21530105 PMC3153580

[16] Chan YY, Yang SN, Lin JC, Chang JL, Lin JG, Lo WY. Inflammatory response in heroin addicts undergoing methadone maintenance treatment. Psychiatry Res. 2015;226(1):230–4. 10.1016/j.psychres.2014.12.053 . 25660662

[17] Chen SL, Lee SY, Tao PL, Chang YH, Chen SH, Chu CH, et al Dextromethorphan attenuated inflammation and combined opioid use in humans undergoing methadone maintenance treatment. J Neuroimmune Pharmacol. 2012;7(4):1025–33. 10.1007/s11481-012-9400-1 . 22990619

[18] Tomescu C, Duh FM, Lanier MA, Kapalko A, Mounzer KC, Martin MP, et al Increased plasmacytoid dendritic cell maturation and natural killer cell activation in HIV-1 exposed, uninfected intravenous drug users. AIDS. 2010;24(14):2151–60. 10.1097/QAD.0b013e32833dfc20 20647906 PMC3253656

[19] Baral S, Sherman SG, Millson P, Beyrer C. Vaccine immunogenicity in injecting drug users: a systematic review. Lancet Infect Dis. 2007;7(10):667–74. 10.1016/S1473-3099(07)70237-2 . 17897609

[20] Kamath GR, Shah DP, Hwang LY. Immune response to hepatitis B vaccination in drug using populations: a systematic review and meta-regression analysis. Vaccine. 2014;32(20):2265–74. 10.1016/j.vaccine.2014.02.072 . 24631093

[21] Corey L, Gilbert PB, Tomaras GD, Haynes BF, Pantaleo G, Fauci AS. Immune correlates of vaccine protection against HIV-1 acquisition. Sci Transl Med. 2015;7(310):310rv7 10.1126/scitranslmed.aac7732 . 26491081 PMC4751141

[22] Rerks-Ngarm S, Pitisuttithum P, Nitayaphan S, Kaewkungwal J, Chiu J, Paris R, et al Vaccination with ALVAC and AIDSVAX to prevent HIV-1 infection in Thailand. N Engl J Med. 2009;361(23):2209–20. 10.1056/NEJMoa0908492 . 19843557

[23] Robb ML, Rerks-Ngarm S, Nitayaphan S, Pitisuttithum P, Kaewkungwal J, Kunasol P, et al Risk behaviour and time as covariates for efficacy of the HIV vaccine regimen ALVAC-HIV (vCP1521) and AIDSVAX B/E: a post-hoc analysis of the Thai phase 3 efficacy trial RV 144. Lancet Infect Dis. 2012;12(7):531–7. 10.1016/S1473-3099(12)70088-9 22652344 PMC3530398

[24] Pitisuttithum P, Gilbert P, Gurwith M, Heyward W, Martin M, van Griensven F, et al Randomized, double-blind, placebo-controlled efficacy trial of a bivalent recombinant glycoprotein 120 HIV-1 vaccine among injection drug users in Bangkok, Thailand. J Infect Dis. 2006;194(12):1661–71. 10.1086/508748 . 17109337

[25] Flynn NM, Forthal DN, Harro CD, Judson FN, Mayer KH, Para MF, et al Placebo-controlled phase 3 trial of a recombinant glycoprotein 120 vaccine to prevent HIV-1 infection. J Infect Dis. 2005;191(5):654–65. 10.1086/428404 . 15688278

[26] Yates NL, Liao HX, Fong Y, deCamp A, Vandergrift NA, Williams WT, et al Vaccine-induced Env V1-V2 IgG3 correlates with lower HIV-1 infection risk and declines soon after vaccination. Sci Transl Med. 2014;6(228):228ra39 10.1126/scitranslmed.3007730 24648342 PMC4116665

[27] Harris PA, Taylor R, Thielke R, Payne J, Gonzalez N, Conde JG. Research electronic data capture (REDCap)—a metadata-driven methodology and workflow process for providing translational research informatics support. J Biomed Inform. 2009;42(2):377–81. 10.1016/j.jbi.2008.08.010 18929686 PMC2700030

[28] Kobie JJ, Zheng B, Bryk P, Barnes M, Ritchlin CT, Tabechian DA, et al Decreased influenza-specific B cell responses in rheumatoid arthritis patients treated with anti-tumor necrosis factor. Arthritis Res Ther. 2011;13(6):R209 10.1186/ar3542 22177419 PMC3334662

[29] Kobie JJ, Alcena DC, Zheng B, Bryk P, Mattiacio JL, Brewer M, et al 9G4 autoreactivity is increased in HIV-infected patients and correlates with HIV broadly neutralizing serum activity. PLoS One. 2012;7(4):e35356 10.1371/journal.pone.0035356 22530008 PMC3329433

[30] Todd DJ, Knowlton N, Amato M, Frank MB, Schur PH, Izmailova ES, et al Erroneous augmentation of multiplex assay measurements in patients with rheumatoid arthritis due to heterophilic binding by serum rheumatoid factor. Arthritis Rheum. 2011;63(4):894–903. 10.1002/art.30213 . 21305505

[31] Bandaru VVR, Mielke M. M., Sacktor N., McArthur J.C., Grant I., Letendre S., Chang L., Wojna V., Pardo C., Calabresi P., Munsaka S., Haughey N.J.. A lipid storage-like disorder contributes to cognitive decline in HIV-infected subjects. Neurology. 2013;In press. 10.1212/WNL.0b013e3182a9565e PMC3888167 24027056

[32] Mielke MM, Bandaru VV, Han D, An Y, Resnick SM, Ferrucci L, et al Demographic and clinical variables affecting mid- to late-life trajectories of plasma ceramide and dihydroceramide species. Aging cell. 2015 10.1111/acel.12369 . 26193443 PMC4693456

[33] Guyon I, Elisseeff. An introduction to variable and feature selection. J Mach Learn Res. 2003;3:1157–82.

[34] Kohavi R, John G. Wrappers for feature subset selection. Artif Intell. 1997;97:273–324.

[35] Guyon I, Weston J., Barnhill S., Vapnik V. Gene selection for cancer classification using support vector machines. Mach Learn. 2002;46:389–422.

[36] Hidalgo-Munoz AR, Ramirez J, Gorriz JM, Padilla P. Regions of interest computed by SVM wrapped method for Alzheimer's disease examination from segmented MRI. Front Aging Neurosci. 2014;6:20 10.3389/fnagi.2014.00020 24634656 PMC3929832

[37] Jourdan M, Caraux A, Caron G, Robert N, Fiol G, Reme T, et al Characterization of a transitional preplasmablast population in the process of human B cell to plasma cell differentiation. J Immunol. 2011;187(8):3931–41. 10.4049/jimmunol.1101230 . 21918187

[38] Fink K. Origin and Function of Circulating Plasmablasts during Acute Viral Infections. Front Immunol. 2012;3:78 10.3389/fimmu.2012.00078 22566959 PMC3341968

[39] Serre K, Cunningham AF, Coughlan RE, Lino AC, Rot A, Hub E, et al CD8 T cells induce T-bet-dependent migration toward CXCR3 ligands by differentiated B cells produced during responses to alum-protein vaccines. Blood. 2012;120(23):4552–9. 10.1182/blood-2012-03-417733 . 23065152

[40] Liu RX, Wei Y, Zeng QH, Chan KW, Xiao X, Zhao XY, et al Chemokine (C-X-C motif) receptor 3-positive B cells link interleukin-17 inflammation to protumorigenic macrophage polarization in human hepatocellular carcinoma. Hepatology. 2015;62(6):1779–90. 10.1002/hep.28020 . 26235097

[41] Henneken M, Dorner T, Burmester GR, Berek C. Differential expression of chemokine receptors on peripheral blood B cells from patients with rheumatoid arthritis and systemic lupus erythematosus. Arthritis Res Ther. 2005;7(5):R1001–13. 10.1186/ar1776 16207316 PMC1257429

[42] Kaminski DA, Wei C, Qian Y, Rosenberg AF, Sanz I. Advances in human B cell phenotypic profiling. Front Immunol. 2012;3:302 10.3389/fimmu.2012.00302 23087687 PMC3467643

[43] Vidarsson G, Dekkers G, Rispens T. IgG subclasses and allotypes: from structure to effector functions. Front Immunol. 2014;5:520 10.3389/fimmu.2014.00520 25368619 PMC4202688

[44] Davidson DC, Jackson JW, Maggirwar SB. Targeting platelet-derived soluble CD40 ligand: a new treatment strategy for HIV-associated neuroinflammation? J Neuroinflammation. 2013;10:144 10.1186/1742-2094-10-144 24289660 PMC3906985

[45] Dwyer R, Topp L, Maher L, Power R, Hellard M, Walsh N, et al Prevalences and correlates of non-viral injecting-related injuries and diseases in a convenience sample of Australian injecting drug users. Drug Alcohol Depend. 2009;100(1–2):9–16. 10.1016/j.drugalcdep.2008.08.016 . 19013725

[46] Ancuta P, Kamat A, Kunstman KJ, Kim EY, Autissier P, Wurcel A, et al Microbial translocation is associated with increased monocyte activation and dementia in AIDS patients. PLoS One. 2008;3(6):e2516 10.1371/journal.pone.0002516 18575590 PMC2424175

[47] Bikman BT, Summers SA. Ceramides as modulators of cellular and whole-body metabolism. J Clin Invest. 2011;121(11):4222–30. 10.1172/JCI57144 22045572 PMC3204836

[48] Kakazu E, Mauer AS, Yin M, Malhi H. Hepatocytes Release Ceramide-enriched Proinflammatory Extracellular Vesicles in an IRE1alpha-dependent Manner. J Lipid Res. 2015 10.1194/jlr.M063412 26621917 PMC4727419

[49] Hammad SM, Pierce JS, Soodavar F, Smith KJ, Al Gadban MM, Rembiesa B, et al Blood sphingolipidomics in healthy humans: impact of sample collection methodology. J Lipid Res. 2010;51(10):3074–87. 10.1194/jlr.D008532 20660127 PMC2936747

[50] Goldkorn T, Chung S, Filosto S. Lung cancer and lung injury: the dual role of ceramide. Handb Exp Pharmacol. 2013;(216):93–113. 10.1007/978-3-7091-1511-4_5 23563653 PMC4370279

[51] Hannun YA. Functions of ceramide in coordinating cellular responses to stress. Science. 1996;274(5294):1855–9. . 8943189 10.1126/science.274.5294.1855

[52] Rivera IG, Ordonez M, Presa N, Gomez-Larrauri A, Simon J, Trueba M, et al Sphingomyelinase D/ceramide 1-phosphate in cell survival and inflammation. Toxins (Basel). 2015;7(5):1457–66. 10.3390/toxins7051457 25938271 PMC4448157

[53] Hernandez-Corbacho MJ, Canals D, Adada MM, Liu M, Senkal CE, Yi JK, et al Tumor Necrosis Factor-alpha (TNFalpha)-induced Ceramide Generation via Ceramide Synthases Regulates Loss of Focal Adhesion Kinase (FAK) and Programmed Cell Death. J Biol Chem. 2015;290(42):25356–73. 10.1074/jbc.M115.658658 26318452 PMC4646185

[54] Regan PM, Dave RS, Datta PK, Khalili K. Epigenetics of micro-opioid receptors: intersection with HIV-1 infection of the central nervous system. J Cell Physiol. 2012;227(7):2832–41. 10.1002/jcp.24004 22034138 PMC3971722

[55] Toskulkao T, Pornchai R, Akkarapatumwong V, Vatanatunyakum S, Govitrapong P. Alteration of lymphocyte opioid receptors in methadone maintenance subjects. Neurochem Int. 2010;56(2):285–90. 10.1016/j.neuint.2009.10.013 . 19913582

[56] Kiebala M, Singh MV, Piepenbrink MS, Qiu X, Kobie JJ, Maggirwar SB. Platelet Activation in Human Immunodeficiency Virus Type-1 Patients Is Not Altered with Cocaine Abuse. PLoS One. 2015;10(6):e0130061 10.1371/journal.pone.0130061 26076359 PMC4467977

[57] Narvaez JC, Magalhaes PV, Fries GR, Colpo GD, Czepielewski LS, Vianna P, et al Peripheral toxicity in crack cocaine use disorders. Neurosci Lett. 2013;544:80–4. 10.1016/j.neulet.2013.03.045 . 23597759

[58] Pereira J, Saez CG, Pallavicini J, Panes O, Pereira-Flores K, Cabreras MJ, et al Platelet activation in chronic cocaine users: effect of short term abstinence. Platelets. 2011;22(8):596–601. 10.3109/09537104.2011.578181 . 21806491

[59] Bagasra O, Forman L. Functional analysis of lymphocytes subpopulations in experimental cocaine abuse. I. Dose-dependent activation of lymphocyte subsets. Clin Exp Immunol. 1989;77(2):289–93. 2528433 PMC1541992

[60] Tomescu C, Seaton KE, Smith P, Taylor M, Tomaras GD, Metzger DS, et al Innate activation of MDC and NK cells in high-risk HIV-1-exposed seronegative IV-drug users who share needles when compared with low-risk nonsharing IV-drug user controls. J Acquir Immune Defic Syndr. 2015;68(3):264–73. 10.1097/QAI.0000000000000470 25514793 PMC4329050

[61] Brenchley JM, Price DA, Schacker TW, Asher TE, Silvestri G, Rao S, et al Microbial translocation is a cause of systemic immune activation in chronic HIV infection. Nat Med. 2006;12(12):1365–71. 10.1038/nm1511 . 17115046

[62] Wallet MA, Rodriguez CA, Yin L, Saporta S, Chinratanapisit S, Hou W, et al Microbial translocation induces persistent macrophage activation unrelated to HIV-1 levels or T-cell activation following therapy. AIDS. 2010;24(9):1281–90. 10.1097/QAD.0b013e328339e228 20559035 PMC2888494

[63] Jiang W, Lederman MM, Hunt P, Sieg SF, Haley K, Rodriguez B, et al Plasma levels of bacterial DNA correlate with immune activation and the magnitude of immune restoration in persons with antiretroviral-treated HIV infection. J Infect Dis. 2009;199(8):1177–85. 10.1086/597476 19265479 PMC2728622

[64] Vassallo M, Mercie P, Cottalorda J, Ticchioni M, Dellamonica P. The role of lipopolysaccharide as a marker of immune activation in HIV-1 infected patients: a systematic literature review. Virol J. 2012;9:174 10.1186/1743-422X-9-174 22925532 PMC3495848

[65] Palmer CD, Tomassilli J, Sirignano M, Romero-Tejeda M, Arnold KB, Che D, et al Enhanced immune activation linked to endotoxemia in HIV-1 seronegative MSM. AIDS. 2014;28(14):2162–6. 10.1097/QAD.0000000000000386 25003719 PMC4460793

[66] Cheng JM, Suoniemi M, Kardys I, Vihervaara T, de Boer SP, Akkerhuis KM, et al Plasma concentrations of molecular lipid species in relation to coronary plaque characteristics and cardiovascular outcome: Results of the ATHEROREMO-IVUS study. Atherosclerosis. 2015;243(2):560–6. 10.1016/j.atherosclerosis.2015.10.022 . 26523994

[67] Reichel M, Honig S, Liebisch G, Luth A, Kleuser B, Gulbins E, et al Alterations of plasma glycerophospholipid and sphingolipid species in male alcohol-dependent patients. Biochim Biophys Acta. 2015;1851(11):1501–10. 10.1016/j.bbalip.2015.08.005 . 26291032

[68] Bergman BC, Brozinick JT, Strauss A, Bacon S, Kerege A, Bui HH, et al Serum sphingolipids: relationships to insulin sensitivity and changes with exercise in humans. Am J Physiol Endocrinol Metab. 2015;309(4):E398–408. 10.1152/ajpendo.00134.2015 26126684 PMC4537923

[69] Larsen PJ, Tennagels N. On ceramides, other sphingolipids and impaired glucose homeostasis. Mol Metab. 2014;3(3):252–60. 10.1016/j.molmet.2014.01.011 24749054 PMC3986510

[70] Mielke MM, Maetzler W, Haughey NJ, Bandaru VV, Savica R, Deuschle C, et al Plasma ceramide and glucosylceramide metabolism is altered in sporadic Parkinson's disease and associated with cognitive impairment: a pilot study. PLoS One. 2013;8(9):e73094 10.1371/journal.pone.0073094 24058461 PMC3776817

[71] Bandaru VV, Mielke MM, Sacktor N, McArthur JC, Grant I, Letendre S, et al A lipid storage-like disorder contributes to cognitive decline in HIV-infected subjects. Neurology. 2013;81(17):1492–9. 10.1212/WNL.0b013e3182a9565e 24027056 PMC3888167

[72] Gracia-Garcia P, Rao V, Haughey NJ, Ratnam Banduru VV, Smith G, Rosenberg PB, et al Elevated plasma ceramides in depression. The Journal of neuropsychiatry and clinical neurosciences. 2011;23(2):215–8. 10.1176/appi.neuropsych.23.2.215 21677254 PMC3121176

[73] Mielke MM, Haughey NJ, Bandaru VV, Weinberg DD, Darby E, Zaidi N, et al Plasma Sphingomyelins are Associated with Cognitive Progression in Alzheimer's Disease. J Alzheimers Dis. 2011 Epub 2011/08/16. D327536MH4R6M204 [pii] 10.3233/JAD-2011-110405 . 21841258 PMC3218198

[74] Machowska A, Carrero JJ, Lindholm B, Stenvinkel P. Therapeutics targeting persistent inflammation in chronic kidney disease. Transl Res. 2016;167(1):204–13. 10.1016/j.trsl.2015.06.012 . 26173187

[75] Leung-Hagesteijn C, Erdmann N, Cheung G, Keats JJ, Stewart AK, Reece DE, et al Xbp1s-negative tumor B cells and pre-plasmablasts mediate therapeutic proteasome inhibitor resistance in multiple myeloma. Cancer Cell. 2013;24(3):289–304. 10.1016/j.ccr.2013.08.009 24029229 PMC4118579

[76] Cheng GL, Zeng H, Leung MK, Zhang HJ, Lau BW, Liu YP, et al Heroin abuse accelerates biological aging: a novel insight from telomerase and brain imaging interaction. Transl Psychiatry. 2013;3:e260 10.1038/tp.2013.36 23695235 PMC3669923

[77] Kovacs GG, Horvath MC, Majtenyi K, Lutz MI, Hurd YL, Keller E. Heroin abuse exaggerates age-related deposition of hyperphosphorylated tau and p62-positive inclusions. Neurobiol Aging. 2015;36(11):3100–7. 10.1016/j.neurobiolaging.2015.07.018 26254956 PMC4609594

[78] Anthony IC, Norrby KE, Dingwall T, Carnie FW, Millar T, Arango JC, et al Predisposition to accelerated Alzheimer-related changes in the brains of human immunodeficiency virus negative opiate abusers. Brain. 2010;133(Pt 12):3685–98. 10.1093/brain/awq263 . 21126996

[79] Reece AS, Hulse GK. Duration of opiate exposure as a determinant of arterial stiffness and vascular age in male opiate dependence: a longitudinal study. J Clin Pharm Ther. 2014;39(2):158–67. 10.1111/jcpt.12121 . 24329809

[80] van Holst RJ, Schilt T. Drug-related decrease in neuropsychological functions of abstinent drug users. Curr Drug Abuse Rev. 2011;4(1):42–56. . 21466500 10.2174/1874473711104010042

[81] Schneider-Schaulies J, Schneider-Schaulies S. Sphingolipids in viral infection. Biol Chem. 2015;396(6–7):585–95. 10.1515/hsz-2014-0273 . 25525752

[82] Cognasse F, Hamzeh-Cognasse H, Lafarge S, Chavarin P, Cogne M, Richard Y, et al Human platelets can activate peripheral blood B cells and increase production of immunoglobulins. Exp Hematol. 2007;35(9):1376–87. 10.1016/j.exphem.2007.05.021 . 17656005

[83] Jego G, Bataille R, Pellat-Deceunynck C. Interleukin-6 is a growth factor for nonmalignant human plasmablasts. Blood. 2001;97(6):1817–22. . 11238125 10.1182/blood.v97.6.1817

[84] Kassambara A, Reme T, Jourdan M, Fest T, Hose D, Tarte K, et al GenomicScape: an easy-to-use web tool for gene expression data analysis. Application to investigate the molecular events in the differentiation of B cells into plasma cells. PLoS Comput Biol. 2015;11(1):e1004077 10.1371/journal.pcbi.1004077 25633866 PMC4310610

[85] McKinney EF, Lee JC, Jayne DR, Lyons PA, Smith KG. T-cell exhaustion, co-stimulation and clinical outcome in autoimmunity and infection. Nature. 2015;523(7562):612–6. 10.1038/nature14468 26123020 PMC4623162

[86] Yao ZQ, Moorman JP. Immune exhaustion and immune senescence: two distinct pathways for HBV vaccine failure during HCV and/or HIV infection. Arch Immunol Ther Exp (Warsz). 2013;61(3):193–201. 10.1007/s00005-013-0219-0 23400275 PMC3792483

[87] Warshow UM, Riva A, Hegazy D, Thurairajah PH, Kaminski ER, Chokshi S, et al Cytokine profiles in high risk injection drug users suggests innate as opposed to adaptive immunity in apparent resistance to hepatitis C virus infection. J Viral Hepat. 2012;19(7):501–8. 10.1111/j.1365-2893.2011.01574.x . 22676363

[88] Leng SX, Dandorf S, Li H, Carlson J, Hui J, Mehta SH, et al Associations of Circulating Soluble Tumor Necrosis Factor-alpha Receptors 1 and 2 with Interleukin-6 Levels in an Aging Cohort of Injection Drug Users with or at High Risk for HIV Infection. AIDS Res Hum Retroviruses. 2015;31(12):1257–64. 10.1089/AID.2015.0134 26414536 PMC4663639

[89] Neri M, Panata L, Bacci M, Fiore C, Riezzo I, Turillazzi E, et al Cytokines, chaperones and neuroinflammatory responses in heroin-related death: what can we learn from different patterns of cellular expression? Int J Mol Sci. 2013;14(10):19831–45. 10.3390/ijms141019831 24084728 PMC3821589

